# The Many Lives of Auranofin: How an Old Anti-Rheumatic Agent May Become a Promising Antimicrobial Drug

**DOI:** 10.3390/antibiotics13070652

**Published:** 2024-07-15

**Authors:** Francesca Coscione, Stefano Zineddu, Valentina Vitali, Marco Fondi, Luigi Messori, Elena Perrin

**Affiliations:** 1 Department of Biology, University of Florence, Via Madonna del Piano 6, I-50019 Sesto Fiorentino, Italy; francesca.coscione@unifi.it (F.C.); marco.fondi@unifi.it (M.F.); 2 Department of Chemistry “Ugo Schiff”, University of Florence, Via della Lastruccia 3-13, I-50019 Sesto Fiorentino, Italy; stefano.zineddu@unifi.it (S.Z.); valentina.vitali@unifi.it (V.V.)

**Keywords:** auranofin, drug repurposing, thioredoxin reductase

## Abstract

Auranofin (AF) is a gold-based compound with a well-known pharmacological and toxicological profile, currently used in the treatment of some severe forms of rheumatoid arthritis. Over the last twenty years, AF has also been repurposed as antiviral, antitumor, and antibacterial drug. In this review we focused on the antibacterial properties of AF, specifically researching the minimal inhibitory concentrations (MIC) of AF in both mono- and diderm bacteria reported so far in literature. AF proves to be highly effective against monoderm bacteria, while diderm are far less susceptible, probably due to the outer membrane barrier. We also reported the current mechanistic hypotheses concerning the antimicrobial properties of AF, although a conclusive description of its antibacterial mode of action is not yet available. Even if its mechanism of action has not been fully elucidated yet and further studies are required to optimize its delivery strategy, AF deserves additional investigation because of its unique mode of action and high efficacy against a wide range of pathogens, which could lead to potential applications in fighting antimicrobial resistance and improving therapeutic outcomes in infectious diseases.

## 1. Introduction

**Figure 1:**
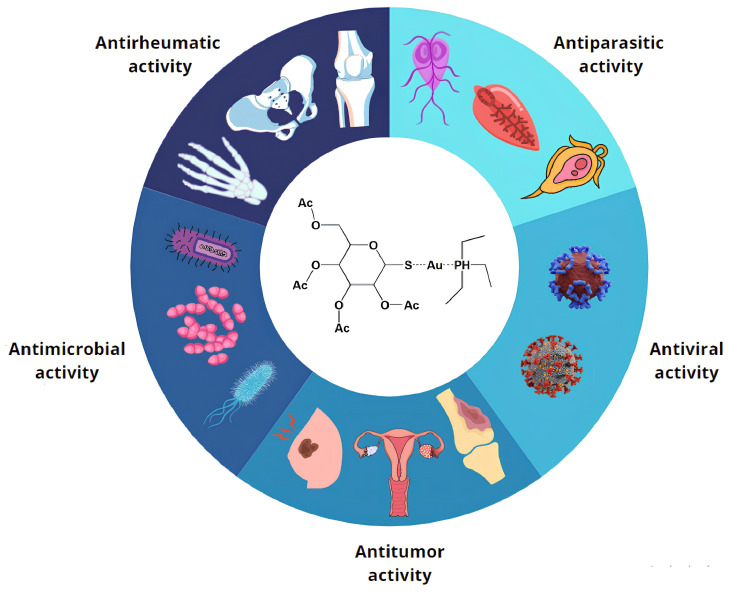
**Auranofin repurposing.** AF has extensive possibilities for clinical applications, supported by experimental evidence gathered from cell lines, animal models, and/or clinical trials. These applications are categorized into five broad groups, reflecting AF’s diverse biological effects and demonstrating its abilities against bacteria, parasites, tumors, rheumatic conditions, and viruses.

Auranofin (AF, CAS 34031-32-8, molecular weight of 678.5 g/mol, Figure 1) is a gold-based compound, approved by the Food and Drug Administration (FDA) in 1985 as an effective anti-rheumatic drug [1,2]. Its chemical structure is based on the presence of a gold(I) center, linearly connected to two distinct substituents, a triethylphosphine ligand on one side and a thiosugar tetraacetate moiety on the other. AF activation typically occurs through release of the thiosugar ligand, making the gold(I) center available for direct interaction with biomolecular targets [3]. It is characterized by a pronounced lipophilic nature and its toxicological properties and safety profile for human treatment are now well-established [4]. This compound is administered orally [5], and it is used in the treatment of juvenile and adult rheumatoid arthritis [6]. In particular, in vitro studies have highlighted its significant inhibitory effects on inflammatory processes [7] and modulation of the human immune system [8].

The last two decades have seen a surge in the so-called “drug repurposing” strategy, which focuses on finding new uses for already approved and established drugs [9]. Due to the difficulties of identifying new antibiotic molecules and the exponential growth of resistant strains to currently used antibiotics, the antimicrobial activity of several approved drugs has already been screened in recent years; for example, phenothiazine neuroleptics and antipsychotic agents [10], local anesthetics [11], antipyretics [12], antihistamines [13], and antihyperlipidemic agents [14]. However, most of these did not demonstrate any antimicrobial activity [15] (Table S1).

On the other hand, metal-based compounds appear to be particularly promising, since they simultaneously act on multiple cellular targets, reducing the possible development of resistance mechanisms [16,17]. However, there are only a few publications on the subject, probably due to the possible toxicity linked to massive use of metals [17]. Among them, the antibacterial activities of Gallium nitrate, approved by FDA for the treatment of cancer-related hypercalcemia, and Cisplatin, an anti-cancer agent, have been tested [18,19,20,21] (Table S2).

AF is a leading example in the application of this approach, with researchers exploring its potential in various therapeutic areas to minimize time loss and financial risks, also considering the mild side effects and the already well known pharmacological and pharmacokinetic profile of this drug [22]. Starting with the identification of possible antiparasitic activity, AF was later assessed for its relevant antiviral, antitumoral, and antibacterial capabilities (Figure 1) [2].

The effective antiparasitic activity of AF has been confirmed against a wide range of organisms [23]. For instance, *Entamoeba histolytica*, a well-known human intestinal parasite, has shown sensitivity to pharmacological treatment with AF in animal models both in vitro and in vivo. Indeed, AF acts by disrupting the proper functioning of the protozoan redox system, ultimately leading to its death [24]. Additionally, research conducted by Peroutka-Bigus and colleagues demonstrated the beneficial effects of AF on *Naegleria fowleri*, a pathogen responsible for a lethal human brain disease, meningoencephalitis [25]. Similarly, *Leishmania donovani* [26] and *Schistosoma mansoni* [27] have also exhibited reduced viability in the presence of this gold-based compound. Finally, successful treatment of mouse models infected with *Giardia lamblia* further confirmed the potential of AF as a therapeutic agent on different types of parasites [28].

Recently, numerous studies have proposed AF as a promising candidate for various antiviral applications. Its ability to inhibit the replication of the human immunodeficiency virus type 1 (HIV-1) and to contribute to the depletion of the viral reservoir in infected patients, as well as that of other RNA viruses, is an appealing feature for the further investigation of its antiviral potential [29]. Moreover, current investigations are actively exploring the antiviral activity of AF against severe acute respiratory syndrome coronavirus 2 (SARS-CoV-2) [7]. K. Sonzogni-Desautels and colleagues hypothesized that future clinical trials may provide promising data, due to Auranofin’s anti-inflammatory properties, which may counteract the so-called “cytokine storm”, a dangerous immune response produced during infection [7].

On the other hand, there is a growing interest in exploring the potential of AF as an antitumoral agent. Several clinical trials have been conducted to investigate its effectiveness in treating acute lymphoblastic leukemia [30] and ovarian [31] and breast tumors in vitro [32]. Although further research is needed to fully understand the clinical mechanism, AF has also been tested as a potential radio modulator in the treatment of colorectal [33] and pancreatic cancer [34].

Beyond the anticancer activity of AF—which, crucially, created new momentum in the investigation of AF as a potential antibacterial agent—is the need for an urgent response to the severe public health problem of antibiotic resistance, combined with an almost complete absence of novel antibiotics [35]. Since developing a new antibiotic from scratch is a highly time consuming and costly process, often resulting in unsuccessful outcomes, screening among existing and approved compounds could greatly speed up the process of finding new eligible candidates for the treatment of bacterial infections [36].

Therefore, the antimicrobial potential of AF has been tested recently [37], both against monoderm and diderm bacteria, producing interesting results, especially towards the former class of pathogens, which could lead to promising and innovative antimicrobial treatments [22,35]. This review aims to summarize the currently available data on the antibacterial activity of AF, providing an overview regarding its efficacy and mechanism of action (MoA). All peer-reviewed articles published in open access from 1997 to 2024 found in established databases were selected.

## 2. Antimicrobial Activity of Auranofin

Based on their cell wall structure, bacteria are usually classified as monoderm or diderm [38]. Monoderm bacteria are characterized by a single cytoplasmic membrane coated by a thick peptidoglycan layer and teichoic or lipoteichoic acids [39]. In contrast, diderm bacteria possess a thinner peptidoglycan layer but a second outer membrane (OM), often containing a lipopolysaccharide (LPS) in the outer leaflet [38]. Despite this classification not being fully representative of the diversity of bacterial cell envelope structures [39] (e.g., in the cell envelope of Mycobacteria and other Actinobacteria, mycolic acid forms a barrier on the outer surface of cells), it is still useful for describing the different behaviors of AF in bacteria.

The first studies exploring the potential repositioning of AF as an antibacterial agent date back to 2009. One of the landmark publications from that year, authored by Jackson-Rosario and colleagues, demonstrated the successful growth inhibition of four strains of *Clostridium difficile* [40] treated with this drug. Similar findings were reported more recently, using clinical isolates (derived from human patient fecal material) and model strains of *C. difficile*, confirming the inhibitory effects of AF towards this bacterial species at concentrations ranging from 0.25 to 4 μg/mL (Table S3) [41].

In the following years, numerous investigations have been conducted to evaluate the antibacterial properties of AF against a wide range of monoderm bacteria, such as *Bacillus subtilis* [42,43], *Enterococcus faecium* [37,42,43,44,45] and *Enterococcus faecalis* [37,42,45,46], not only on wild-type but also on drug-resistant variants (Table S3).

The activity of AF against tuberculous [42] and non-tuberculous mycobacteria (NTM) (which show high levels of resistance against many commercially available antibiotics) has been investigated [47]. The growth of *Mycobacterium tuberculosis*, responsible for tuberculosis, was inhibited by low concentrations of AF (in the range of 0.5–4 μg/mL) [42,47], while Ruth et al. showed promising minimal inhibitory concentration (MIC) data against NTM, such as *Mycobacterium abscessus*, but not against strains belonging to the *Mycobacterium avium* complex (Table S3) [47].

A comprehensive view of the action of AF against *Staphylococcus aureus* is currently available [43,46]. For example, methicillin-resistant *S. aureus* (MRSA), a pathogen responsible for invasive human diseases, and methicillin-susceptible *S. aureus* (MSSA) were subjected to the gold-based compound treatment, demonstrating significant antimicrobial activity (Table S3) [37,42,44,47,48]. In addition, AF has been tested against several vancomycin-resistant *S. aureus* (VRSA) strains, resulting in MIC values ranging from 0.0625 to 0.5 μg/mL (Table S3) [37,42]. Finally, the growth of glycopeptide-intermediate and vancomycin-intermediate *S. aureus* (VRSA and VISA) were also inhibited in presence of minimal concentrations of AF, as demonstrated by Thangamani and colleagues (Table S3) [37]. Furthermore, the antimicrobial activity of AF was evaluated against a group of different strains and clinical isolates of *Streptococcus*, revealing positive results with MIC values below 0.25 μg/mL for the different species analyzed (Table S3) [37,45,49].

In contrast, in diderms, several studies have indicated (Table S3) a lower sensitivity to the drug compared to monoderms. For example, *Acinetobacter baumannii* was inhibited only by high concentrations of AF, with MIC values well above 50 μg/mL (Table S3) [37,42,44,45,50,51]. Similarly, the growth of *Stenotrophomonas maltophilia* [45], *Enterobacter cloacae* [44,51], *Salmonella typhimurium* [37], and of the model bacterium *Escherichia coli* are inhibited only in the presence of high concentrations of AF (Table S3) [37,44,45,48,50,52,53,54].

Likewise *Klebsiella pneumoniae* (an opportunistic pathogen associated with pneumonia) and *Pseudomonas aeruginosa* (a pathogen responsible for severe antibiotic-resistant infections), showed higher MIC values between 16 and 256 μg/mL [37,42,44,45,50,51] and above 256 μg/mL, respectively (Table S3) [37,42,44,45,48,50,51].

Finally, AF has been tested on the *Burkholderia* genus, a group of diderm bacteria whose members inhabit a wide range of ecological niches, including soil, plant rhizospheres, water, animal species, and humans [55,56]. Maydaniuk et al. also found a low efficacy of AF against these species (Table S3) [56].

Some notable exceptions to this general low activity trend against diderm bacteria have been identified in *Burkholderia mallei* [56], *Helicobacter pylori* [57], *Neisseria gonorrhoeae* [58] and *Bacteroides fragilis* [59], for which the reported MIC values are more in line with the ones observed for monoderms, with values varying between 0.25 and 1 μg/mL (Table S3).

Interestingly, two research teams independently demonstrated a stronger antibacterial activity of AF against diderm bacteria using Polymyxin B nonapeptide hydrochloride (PMBN) [37,45], a permeabilizing agent. When used in combination with AF, this compound triggered a significant reduction in MIC values, thereby improving the susceptibility of diderms (Table S3).

## 3. Mechanism of Action

The observation of significant antimicrobial properties for AF, as detailed above, sparked great attention in the underlying molecular mechanisms (MoA). Accordingly, several studies addressed the expected mechanisms responsible for the antimicrobial properties of AF.

As mentioned above, the first studies on the repositioning of AF as an antimicrobial concerned its potential antiparasitic activity and coincided with those regarding its MoA. AF turned out to be a potent in vitro inhibitor of selenoproteins, such as the thioredoxin reductase enzyme (TrxR) in *E. histolytica* [24,60] and thioredoxin–glutathione reductase (TGR) in *S. mansoni* [27,61]. Inhibition of TrxR produced by AF in these microorganisms is mainly ascribed to direct gold association with functional cysteines on the active site of the enzyme.

**Figure 2:**
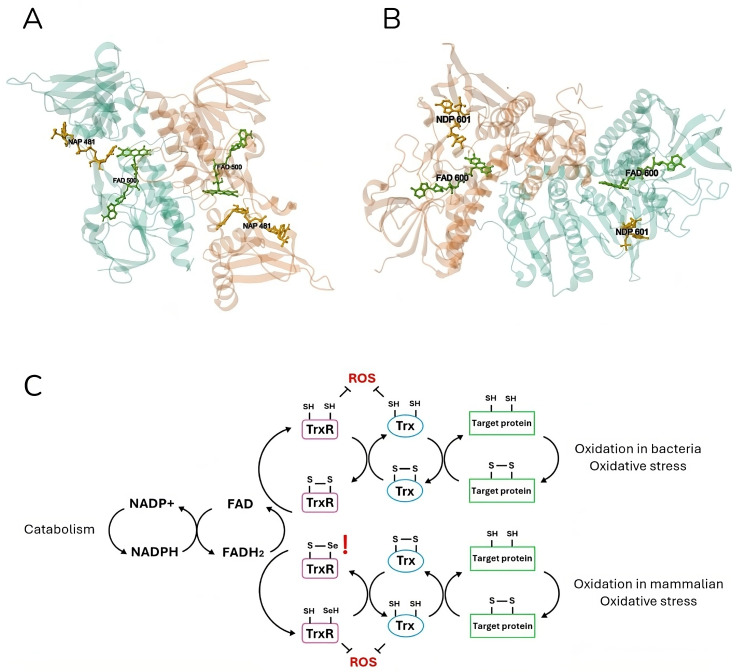
**Crystal structure and mechanism of mammalian and bacterial thioredoxin reductase**. *Escherichia coli* (**A**) (PDB DOI: https://doi.org/10.2210/pdb1TDF/pdb [63]) and (**B**) *Rattus norvegicus* (https://doi.org/10.2210/pdb1H6V/pdb [64]) thioredoxin reductases are shown in ribbon representation. Both homodimer enzymes contain a FAD prosthetic group (green) and an NADPH binding domain (yellow), shown in ball-and-stick representation, and an active site with a redox-active disulfide bond. The electron flow in the Trx system is illustrated (**C**), respectively, for the bacterial (up) and mammalian (down) catabolism. Thioredoxin reductase (TrxR) is reduced by FADH_2_ using electrons from NADPH. The latter in turn reduces thioredoxin (Trx) by oxidizing it. Finally, Trx reduces target proteins before being reduced again by TrxR. Both mammalian and bacteria active sites are shown in detail (**C**). In eukaryotic cells, TrxR specifically contains a highly nucleophilic selenium (Se) atom in the form of selenocysteine. TrxR and Trx can also directly reduce ROS, providing the cell with a strong defense system.

Indeed, TrxR is a nearly ubiquitous enzyme that is present both in eukaryotic and prokaryotic systems. Two different classes of this enzyme have evolved independently: higher eukaryotic organisms, including humans, possess a higher molecular weight (55 kDa per subunit) selenocystein-containing isoform of the enzyme, whereas prokaryotic organisms present a lower molecular weight variant (35 kDa per subunit) and normally lack the presence of this amino acid in their active site (Figure 2). Additionally, these two proteins differ in terms of their electron transfer reaction: while the eukaryotic TrxR is characterized by two sequentially involved active sites—one at the FAD binding domain and one at the C-terminal (the latter containing the selenocysteine residue)—, the prokaryotic one possesses a single active site at the NADPH binding domain [49]. So, the bacterial TrxR is a 70-KDa homodimeric flavoenzyme which possesses a redox active site and a catalytic site on each of its subunits. While the former site hosts a molecule of flavin adenine dinucleotide (FAD), the latter is composed of a CXXC aminoacidic motif [62].

TrxR is part of the thioredoxin (Trx) system, which employs NADPH to reduce disulfide bonds on cytoplasmic enzymes, thereby regulating many intracellular processes including redox homeostasis, DNA synthesis, and detoxification from xenobiotics, oxidants, and radicals [49,65,66]. The same functions can be performed by the glutaredoxin (Grx) system (NADPH, glutathione reductase (GR), glutathione (GSH), and Grx) [67]. TGR is an enzyme found in many organisms, capable of combining the activities of TrxR and GR into a single protein [68].

Based on these previous data on parasites, literature on the MoA in bacteria focused on TrxR as the main—although not the only—target of AF. As previously discussed, the global MIC values clearly indicate that, in general, AF demonstrates more potent antimicrobial activity against monoderm rather than diderm bacteria. This disparity in efficacy suggests that AF interacts differently with monolayer and bilayer bacteria, potentially indicating variations in its mechanism of action. The following two sections will address the most established theories regarding AF’s MoA in more depth, highlighting the differences between these two categories of bacteria.

### 3.1. Monoderm Bacteria

Initially, the bacterial selenium metabolism has been suggested as the most likely target of AF. One of the earliest investigations was carried out in *C. difficile*, where the presence of AF was linked to disruption of the selenium metabolism by directly preventing this element’s uptake from the bacterial growth medium [40]. Since selenium is a crucial micronutrient in the biosynthetic pathway of selenoproteins through its incorporation into selenocysteine residues, one of the first hypotheses was that the lack of selenium could impair the biosynthesis of these proteins, which could ultimately lead to inhibition of cell growth [40]. However, this hypothesis has been lately proven incorrect since *C. difficile* strains lacking selenoproteins were as susceptible to AF as their respective wild-type strains [69]. In addition, they demonstrated that selenite supplementation reduces the activity of AF both in the presence and absence of selenoproteins. This suggested that selenite’s neutralization of AF is not due to a compensation for a chemically induced selenium deficiency [69]. 

**Figure 3:**
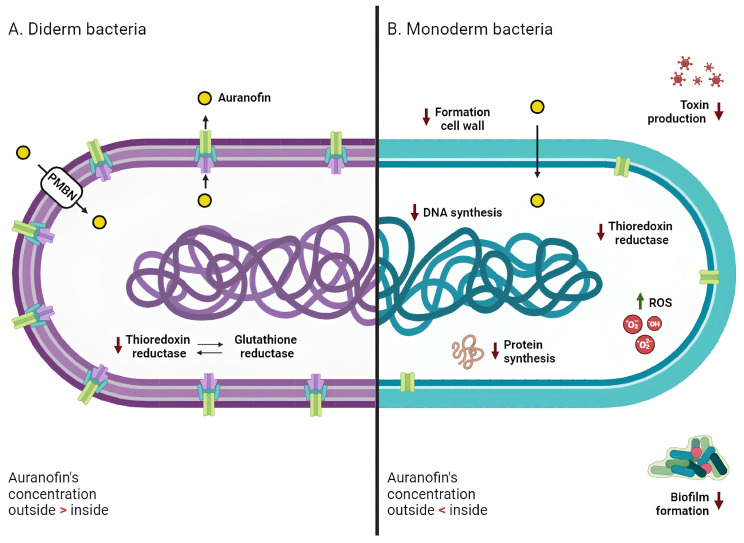
**Mechanism of action of Auranofin against diderm and monoderm bacteria.** In both bacterial types (**A** for diderm and **B** for monoderm), the primary target of AF’s action is thioredoxin reductase, whose inhibition promotes an intracellular increase in reactive oxygen species (ROS) and subsequent oxidative stress. In monoderm bacteria, AF also inhibits different pathways, such as DNA, protein, and cell wall synthesis. AF demonstrates antibiofilm properties and is able to counteract bacterial toxin production. Diderm bacteria exhibit lower sensitivity to AF; this resistance may be attributed to the presence of glutathione reductase (compensating for the lack of TrxR activity), the outer membrane (PMBN, a permeabilizing agent, promotes AF entry into the cells), and efflux pumps (actively pushing drug molecules into the extracellular space). Red arrows indicate a decrease, and green arrows indicate an increase.

As mentioned above, from this point onwards, AF has been extensively tested against a huge panel of monoderm bacteria, including MSRA, MSSA, VRSA, and VISA, and the collected results suggest that the main target of the growth inhibitory action is TrxR (Figure 3) [42,70,71]. The mechanism involved in this inhibition likely occurs through a displacement of the most labile ligand of AF (the thiosugar moiety) from the gold center, with the subsequent formation of a novel bond between the metal and the thiol group of the cysteine residue in the TrxR active site. As TrxR is an essential gene that regulates bacterial thiol-redox homeostasis, antibacterial treatment with AF induces oxidative stress and depletion of thiols in the cell [72]. In particular, TrxR is essential for DNA synthesis and protein repair through the reduction of ribonucleotide and methionine sulfoxide reductase, respectively [42].

Since *S. aureus* does not develop resistance against AF, this compound may have more than one intracellular target, encouraging the exploration of alternative mechanisms of action of AF in bacteria [71]. In support of this theory, Thangamani and colleagues have shown that the antibacterial action of this gold-based compound can be extremely complex, involving the inhibition of biosynthetic pathways including DNA, protein synthesis, and cell wall formation (Figure 3) [37]. In addition, AF suppresses toxin production in *S. aureus*, *S. epidermidis* and *C. difficile* in a dose-dependent manner [37,41], as well as spore formation in *C. difficile* (Figure 3) [41]. This again suggests that the effect of AF may reverberate at the whole cell level and not just affect the direct target of its MoA.

Another remarkable effect of AF has been linked to the reduction of the biofilm mass formed by *S. aureus* and *S. epidermidis* [72,73] Biofilms are aggregates of microorganisms in which cells are frequently embedded in a self-produced matrix of extracellular polymeric substances (EPS) that are adherent to each other and/or to a surface [74]. Growth as a biofilm represents a very widespread lifestyle among microorganisms both in the environment and within a host, and infections caused by microbial biofilms represent a serious clinical challenge [75]. Indeed, protection against the immune system and the action of antibiotics are among the numerous advantages that biofilms offer to bacterial cells [75]. Thus, introducing valuable agents to fight this could be crucial for counteracting bacterial infections. Surprisingly, AF efficacy in this field far surpasses that of the antimicrobial drugs currently exploited for bacterial infection treatments, such as vancomycin and linezolid, showing higher mass reductions at lower administered concentrations [73]. Moreover, AF has shown a positive effect on the planktonic persister cells of *S. aureus* (Figure 3) [76]. Bacterial persistence represents another important clinical challenge and is implicated in the development of chronic infections. Persisters are cells with a reduced metabolism that allow them to transiently display phenotypic tolerance to antibiotics [77]. As in the case of biofilms, AF action against these types of cells represents an important new avenue for the application of this drug.

In summary, the current hypothesis for monoderm bacteria envisions a multitarget mechanism, where the TrxR enzyme is the main target but other alternative pathways could also be affected by exposure to the drug and, overall, lead to growth inhibition.

### 3.2. Diderm Bacteria

Even in the case of diderm bacteria, TrxR has been recognized as the main target of AF, despite the recorded reduced sensitivity to this gold compound with respect to monoderm bacteria (Figure 3) [42,44]. As previously mentioned, the Grx system can operate in parallel with the Trx system [49]. This system has been found to be absent in many pathogenic monoderm bacteria, such as *S. aureus* and *H. pylori*, confirming the essentialness of the Trx system in responding to oxidative stress [67].

The drop in susceptibility of diderms (as compared to monoderms) with respect to AF was initially supported by the simultaneous presence of both systems, as the Grx system compensates for the reduced functionality of TrxR [42]. However, studies have shown that an *E. coli* double mutant strain (Origami-2), containing mutations in both the thioredoxin reductase (*trxB*) and glutathione reductase (*gor*) genes, did not display a reduced growth pattern in the presence of AF compared to the wild type strain [37]. This is also in line with the recent proposal that glutathione reductase in *B. cenocepacia* is not a target of AF [56].

The currently accepted hypothesis is that the outer membrane of diderm bacteria may contribute significantly to their lower susceptibility to the drug [37]. Exposure of bacteria to a combination of AF and a permeabilizing agent, such as PMBN, resulted in significantly lower MICs, ranging from 0.125 to 8 μg/mL [37,45] (Table S3). The efficacy of AF against diderms may be hindered not only by the physical barrier of the outer membrane but also by the presence of efflux pumps, which are predominantly present in diderms [78]. Efflux pumps are membrane proteins that actively pump unwanted substances out of cells; they are crucial in the development of drug resistance by helping cells evade the effects of certain antimicrobial agents or chemicals [79]. These systems may be involved in the surge of resistance mechanisms of diderm bacteria to AF. This theory was confirmed by observing that deletion of the *acrAB* pump in *E. coli*—which was shown to contribute to the antibiotic-resistant phenotype in multiple strains and to be implicated in the resistance to numerous antibiotics including ampicillin, rifampicin, and chloramphenicol—reduced the MIC of AF from 32 to 8 μg/mL [37]. Further research is needed to assess the exact role of efflux pumps in the development of AF resistance/tolerance.

Finally, AF reduces the expression of the *ompA* gene in *B. fragilis*, coding for an important component of the outer membrane involved in several cellular functions, including adhesion to substrate and regulation of cell shape [59]. This suggests that AF may also interfere with biofilm formation and that it could prevent the formation of capsules, the most typical virulence factor of *B. fragilis* [59].

## 4. Conclusions and Perspectives

In summary, AF has been shown to have important antibacterial activity both in vitro and in vivo (higher than many other repurposed drugs) and may thus be a promising candidate for drug repurposing in the treatment of multi-resistant pathogens. Particularly relevant is its action against *E. faecium* and *S. aureus* (including MSRA, MSSA, VRSA, and VISA), which are included in the ESKAPE (acronym for *E. faecium*, *S. aureus*, *K. pneumoniae*, *A. baumanii*, *P. aeruginosa*, and *Enterobacter* species) pathogens panel. ESKAPE bacteria are the main cause of nosocomial infections worldwide and are particularly dangerous due to their high pathogenicity and antibiotic resistance [80]. Likewise, AF action against *M. tuberculosis* and the NTM bacterium *M. abscessus* is of particular interest. Indeed, tuberculosis remains a widespread disease with a high mortality rate, which becomes increasingly difficult to treat due to the progressive ineffectiveness of anti-tuberculosis drugs [81]. On the other hand *M. abscessus* causes severe lung infections in immunocompromised individuals and is difficult to treat owing to its high antibiotics resistance [82].

The mode of action of AF appears to be complex and is not yet fully understood. Undoubtedly, the enzyme thioredoxin reductase remains a crucial target. However, several hypotheses suggesting a multi-target mechanism have been put forward. The combination of AF interference with bacterial redox metabolism, inhibition of bacterial thioredoxin reductase, and intense oxidative stress seem to be the most convincing hypotheses of this compound’s main mechanism. At the same time, there is strong evidence that other targets and other pathways may be involved in determining the actual antimicrobial action of AF. A more precise understanding of the overall mode of action is likely to emerge from multiomics research programs, in which the effect on multiple cellular targets (if any) could be detected in a single experiment. We believe that particular attention should also be paid to AF’s action against persister cells and to its ability to disrupt biofilm formation.

Another interesting point that needs further exploration is the different activity against mono- and diderm bacteria. Diderm bacteria are intrinsically more resistant to antibiotics than monoderm bacteria, thanks to the combined activity of the outer membrane and of efflux pumps located in the cytoplasmic membrane, which prevent antimicrobial accumulation inside the cell [83]. While several studies demonstrated the role of the outer membrane in reducing the AF sensitivity of diderm bacteria, the possible role of efflux pumps is still poorly investigated.

Further studies along these lines will hopefully lead to better identification of the biomolecular targets and to optimization of the metallodrug itself, through rational chemical modifications in the scaffold, which could allow for an increased effectiveness in diderm bacteria. Indeed, to the best of our knowledge, several auranofin analogues that might be comparatively investigated are already available.

Finally, to enhance the potency of antimicrobial treatment, AF could be conveniently incorporated into combination therapies. This kind of approach is a cornerstone methodology for treating tuberculosis (TB), and recent studies have demonstrated that the administration of auranofin together with already exploited anti-TB agents, such as rifampicin and isoniazid, can lead to promising results [42]. Therefore, the compatibility of auranofin with these antituberculotic agents is crucial in developing novel strategies that can hopefully increase the global efficacy of antimicrobial therapies.

## Data Availability

No new data were created or analyzed in this study. Data sharing is not applicable to this article.

## Supplementary Materials

The following supporting information can be downloaded at: https://www.mdpi.com/article/10.3390/antibiotics13070652/s1, Table S1: Antimicrobial activity of some antipsychotics, local anesthetics, antipyretics, antihyperlipidemics, and antihistamines. Table S2: Antimicrobial activity of Gallium nitrate and Cisplatin. Table S3: Minimal inhibitory concentration (MIC, µg/mL) of Auranofin in Monoderm e Diderm bacteria. Abbreviations: MRSA, methicillin-resistant *S. aureus*; MSSA, methicillin-susceptible *S. aureus*; VRSA, vancomycin-resistant *S. aureus*; VISA, vancomycin-intermediate *S. aureus*; PMBN, Polymyxin B nonapeptide hydrochloride.
